# Seroprevalencia de SARS-CoV-2 y factores asociados en migrantes con vocación de permanencia, Bucaramanga, Colombia

**DOI:** 10.15649/cuidarte.2657

**Published:** 2023-03-29

**Authors:** Ruth Aralí Martínez-Vega, Alix Bolívar-Grimaldos, Bladimiro Rincón-Orozco

**Affiliations:** 1 . Universidad de Santander, Escuela de Medicina, Bucaramanga, Colombia. Email: ruth.martinez@udes.edu.co Universidad de Santander Universidad de Santander Colombia ruth.martinez@udes.edu.co; 2 . Laboratorios Bolívar, Bucaramanga, Colombia. Email: alixbolivar@laboratoriobolivar.com Laboratorios Bolívar Colombia alixbolivar@laboratoriobolivar.com; 3 . Universidad Industrial de Santander, Escuela de Medicina, Departamento de Ciencias Básicas, Bucaramanga, Colombia. Email: blrincon@uis.edu.co Universidad Industrial de Santander Universidad Industrial de Santander Colombia blrincon@uis.edu.co

**Keywords:** SARS-CoV-2, Estudios Seroepidemiológicos, Migrantes., SARS-CoV-2, Seroepidemiologic Studies, Transients and Migrants., SARS-CoV-2, Estudos Soroepidemiológicos, Migrantes.

## Abstract

**Introducción:**

Existen pocos reportes que evalúen la seroprevalencia contra SARS-CoV-2 en población migrante en el mundo. Estos estudios ayudan a conocer la exposición al virus en las poblaciones para implementar acciones que reduzcan el impacto de la infección por SARS-CoV-2.

**Objetivo:**

Determinar la seroprevalencia contra SARS CoV-2 en migrantes con vocación de permanencia en Bucaramanga, e identificar factores asociados a la infección previa por SARS-CoV-2.

**Materiales y métodos:**

Estudio de corte transversal analítico con muestreo consecutivo. Se incluyeron migrantes adultos en Bucaramanga durante febrero/2021. Se realizaron encuestas e inmunoensayos de quimioluminiscencia para IgM e IgG contra SARS-CoV-2 en suero. Se calcularon razones de prevalencia (RP) con regresión log-binomial.

**Resultados:**

Se incluyeron 462 participantes. La seroprevalencia de IgM fue 11,7% (IC95% 9,1-14,9), de IgG fue 32,9% (IC95% 28,8 37,3) y de IgM o IgG fue 36,1% (IC95% 31,9-40,6). Exposición a un caso confirmado (RP:1,54; IC95%1,04-2,29) o sospechoso (RP:1,56; IC95%1,13-2,16) de COVID-19, seis o más convivientes (RP:1,52; IC95%1,05-2,20), estancia en Colombia >2 años (RP:1,43; IC95%1,11-1,92) y presencia de síntomas (RP:1,62; IC95%1,26 - 2,10) se asociaron con mayor seroprevalencia de IgG.

**Discusión:**

En Bucaramanga, la seroprevalencia en migrantes fue similar a la de migrantes en Kuwait, pero menor que en Paris y Singapur.

**Conclusión:**

En migrantes con vocación de permanencia la seroprevalencia contra SARS-CoV-2 fue similar a la reportada en residentes de Bucaramanga. El contacto con casos sospechosos/confirmados de COVID-19 y las condiciones de hacinamiento fueron algunos de los factores asociados a la seroprevalencia.

## Abstract (en)

**Introduction:**

There are few reports assessing anti-SARS-CoV-2 seroprevalence in the migrant population in the world. These studies help to understand the exposure of populations to the virus to take actions to reduce the impact of SARS-CoV-2 infection.

**Objective:**

To determine the anti-SARS-CoV-2 seroprevalence in migrants with a vocation for permanence in Bucaramanga and to identify factors associated with previous SARS-CoV-2 infection.

**Methods:**

Analytical cross-sectional study using consecutive sampling, which included adult migrants in Bucaramanga in February 2021. Surveys were conducted, and chemiluminescent immunoassays were performed to detect IgM and IgG antibodies to SARS-CoV-2 in serum samples. Prevalence ratios (PR) were estimated using a log-binomial regression model.

**Results:**

The study enrolled 462 participants. IgM seroprevalence was 11.7% (95% CI 9.1-14.9), IgG seroprevalence was 32.9% (95% CI 28.8-37.3), and IgM or IgG seroprevalence was 36.1% (95% CI 31.9-40.6). Contact with a confirmed case of COVID-19 (PR:1.54; 95% CI 1.04-2.29) or suspected case (PR:1.56; 95% CI 1.13-2.16); living with six or more people (PR: 1.52; 95% CI 1.05-2.20); stay in Colombia > 2 years (PR:1.43; 95% CI 1.11-1.92), and presence of symptoms (PR:1.62; 95%CI 1.26-2.10) were some factors associated with higher IgG seroprevalence.

**Discussion:**

In Bucaramanga, SARS-CoV-2 seroprevalence among migrants was similar to the seroprevalences of migrants in Kuwait but lower than migrants in Paris and Singapore.

**Conclusions:**

The anti-SARS-CoV-2 seroprevalence among migrants with a vocation for permanence was similar to that reported among residents of Bucaramanga. Contact with suspected or confirmed COVID-19 cases and crowded conditions were some of the factors associated with seroprevalence.

## Resumo

**Introdujo:**

Existem poucos relatos que avaliam a soroprevalencia contra SARS-CoV-2 na populagáo migrante no mundo. Esses estudos ajudam a conhecer a exposigáo ao virus nas populates para implementar agóes que reduzam o impacto da infecgáo por SARS-CoV-2.

**Objetivo:**

Determinar a soroprevalencia contra SARS CoV-2 em migrantes que pretendem permanecer em Bucaramanga e identificar fatores associados a infecgáo anterior por SARS-CoV-2.

**Métodos:**

Estudo analítico transversal com amostragem consecutiva. Foram incluídos migrantes adultos em Bucaramanga durante fevereiro/2021. Foram realizados levantamentos e imunoensaios de quimioluminescencia para IgM e IgG contra SARS-CoV-2 no soro. As razóes de prevalencia (RP) foram calculadas com regressáo log-binomial.

**Resultados:**

foram incluídos 462 participantes. A soroprevalencia de IgM foi de 11,7% (IC 95% 9,1-14,9), de IgG foi de 32,9% (IC 95% 28,8 37,3) e de IgM ou IgG foi de 36,1% (IC95% 31,9-40,6). Exposigáo a um caso confirmado (RP: 1,54; IC 95% 1,04-2,29) ou caso suspeito (RP: 1,56; IC 95% 1,13-2,16) de COVID-19, seis ou mais coabitantes (RP: 1,52; IC 95% 1,05 -2,20), permanencia na Colombia >2 anos (Rp: 1,43; IC 95% 1,11-1,92) e presenga de sintomas (RP: 1,62; IC95%1,26 - 2,10) foram associados a maior soroprevalencia de IgG.

**Discussao:**

Em Bucaramanga, a soroprevalencia em migrantes foi semelhante a de migrantes no Kuwait, mas menor do que em Paris e Cingapura.

**Conclusao:**

Em migrantes com vocagáo de permanencia, a soroprevalencia contra SARS-CoV-2 foi semelhante a relatada em residentes de Bucaramanga. O contato com casos suspeitos/confirmados de COVID-19 e as condigóes de superlotagáo foram alguns dos fatores associados a soroprevalencia.

## Introducción

El síndrome respiratorio agudo severo pandémico, denominado enfermedad por coronavirus 2019 (COVID-19) es causado por el coronavirus SARS-CoV-2, que produce desde infecciones asintomáticas hasta la muerte^1^. Desde el inicio de la pandemia hasta el 31 de mayo de 2021 Colombia había reportado a la Organización Mundial de la Salud 3.518.046 infecciones confirmadas y 90.890 muertes causadas por este virus^2^. Hasta el 30 de junio de 2020 se habían presentado 1.297 casos en migrantes y refugiados provenientes de Venezuela, de los cuales habían fallecido 1,6%^3^.

La comunidad científica ha realizado un llamado internacional para que la población migrante y refugiada se tenga en cuenta durante la respuesta a COVID-19, incluyendo acceso a diagnóstico, tratamiento y vacunación, puesto que viven y trabajan bajo condiciones que dificultan cumplir las recomendaciones para evitar la transmisión del virus. Además, se encuentran en precariedad económica porque se han reducido o perdido sus ingresos, y tienen barreras de acceso a la salud pública y a los servicios sociales^4^^,^^5^^,^^6^^,^^7^.

Existen pocos reportes que evalúen la seroprevalencia de SARS-CoV-2 en población migrante en el mundo^8^^,^^9^^,^^10^. Por ejemplo en estudios realizados en 2020, uno que incluyó hombres adultos, migrantes, asintomáticos y trabajadores de supermercados en Kuwait, reportó una seroprevalencia de anticuerpos IgG/IgM de 38,1% (IC95% 34,0-42,38). Otro estudio realizado en París, que incluyó una subpoblación de adultos migrantes trabajadores, residentes en dos refugios de Médicos sin Fronteras, reportó una seroprevalencia de anticuerpos IgG de 89% (IC95% 81,8-93,29). Además, en Singapur se encontró una seroprevalencia de 60,1% en hombres adultos, migrantes, trabajadores, asintomáticos y sintomáticos, residentes en edificios-dormitorios^10^.

En cuanto a los factores relacionados con la seroprevalencia en población migrante, se ha reportado una seroprevalencia significativamente menor en fumadores comparados con no fumadores (28,0% vs 44,7%, p<0,0018). Así mismo en el estudio realizado en París, el tabaquismo fue reportado como factor relacionado con menor seroprevalencia en fumadores regulares comparados con no fumadores (ORa 0,4; IC95% 0,3 - 0,7), así como el sexo femenino (ORa 0,5; IC95% 0,4 - 0,8), mientras que el hacinamiento alto (ORa 3,4 IC95% 1,7 - 6,9) y la asistencia a gimnasio (ORa 3,1; IC95% 1,2 - 8,1) se asociaron a mayor seroprevalencia^9^.

Los estudios que evalúan la seroprevalencia ayudan a conocer la exposición al virus en las poblaciones para poder implementar acciones para reducir el impacto de la infección por SARS-CoV-2. Por lo anterior, el objetivo del estudio fue determinar la seroprevalencia de anticuerpos IgG e IgM contra SARS CoV-2 en población migrante con vocación de permanencia en Bucaramanga, así como identificar factores asociados a la infección previa por SARS-CoV-2.

## Materiales y Métodos

### Tipo de estudio, población y muestra:

Esta investigación fue un estudio de corte transversal analítico con muestreo consecutivo. Se incluyeron migrantes con vocación de permanencia, con 18 o más años que acudieron a la Fundación Entre dos Tierras en Bucaramanga, Colombia durante febrero de 2021. Se excluyeron las personas con trastorno cognitivo o mental que impedía otorgar consentimiento informado. No se realizó cálculo del tamaño de muestra porque se invitaron a participar a todos los migrantes adultos, con vocación de permanencia que acudieron durante el periodo de estudio a la Fundación.

### Procedimiento para recolección de la información y variables:

Se realizó una encuesta que incluyó variables demográficas, comorbilidades, tabaquismo, talla y peso para calcular el Índice de Masa Corporal (IMC), presencia de síntomas sugestivos de COVID-19, exposición a casos de COVID-19 sospechosos o confirmados y algunas prácticas relacionadas con la transmisión de SARS-CoV-2. Posteriormente, se tomó temperatura, pulso, oximetría, y muestra de sangre venosa. Estos procedimientos fueron realizados por un equipo de seis profesionales Microbiólogas y Bioanalistas, quienes habían recibido un entrenamiento previo para la aplicación de la encuesta y la toma de signos vitales.

Se realizaron inmunoensayos de quimioluminiscencia para detectar IgM e IgG contra SARS-CoV-2 siguiendo las recomendaciones del fabricante, Snibe, Maglumi 2019-CoV (IgM: sensibilidad 77,5%- 87,5% y especificidad 97,5%-100%; IgG: sensibilidad 87,5%-100% y especificidad entre 97,3%-100%, en sintomáticos de >14 días^11),(^^12^^,^^13^^,^^14^. Se consideró una infección sintomática cuando el participante contestó afirmativamente a la pregunta ¿Ha tenido síntomas de COVID-19? y especificó al menos uno de los siguientes síntomas: cefalea, anosmia, ageusia, tos, fiebre, odinofagia. También se preguntaron disnea, dolor torácico, mialgias, fatiga, diarrea y dolor abdominal, estos se presentaron en compañía de otros síntomas.

### Análisis de la información:

Para el análisis, las variables cualitativas fueron descritas con frecuencias absolutas y relativas, y las cuantitativas con mediana y rango intercuartil (RIQ: Q1-Q3) porque no presentaron distribución normal. Se calcularon las seroprevalencias para IgM e IgG, e intervalos de confianza de 95% (IC95%) utilizando el método de Wilson. Se ajustó la prevalencia considerando sensibilidad y especificidad de la técnica^15^. Además, se realizó un análisis univariado considerando como desenlace la seropositividad para IgG y como exposiciones las características demográficas y clínicas, y antecedentes, usando la prueba exacta de Fisher y U de Mann-Whitney. Para determinar los factores asociados a la seroprevalencia se calcularon las razones de prevalencia (RP) con regresión log-binomial. Los análisis se realizaron en el programa Stata 16.1. La base de datos de este estudio se encuentra disponible en el gestor público Mendeley Data^16^.

### Consideraciones éticas:

Esta investigación fue aprobada por el Comité de Ética en Investigación Científica de la UIS (Acta No. 20 del 27 de noviembre de 2020). Cada participante firmó consentimiento informado.

## Resultados

Entre el 2 y 20 de febrero del 2021 se incluyeron 462 (73,33%) participantes de 630 incluibles que fueron citados a la Fundación. Los participantes eran adultos en edad reproductiva, la mayoría mujeres (7/434; 1,61% embarazadas) sin seguridad social. La mayoría de los participantes habían nacido en Venezuela; solo tres refirieron que habían nacido en Colombia, emigraron a Venezuela e inmigraron recientemente. La mediana de años de estudio fue 11, y sólo 5,19% se consideró desempleado. Además, 50% residen en Colombia desde hace más de 2,05 años, con al menos cinco personas en la vivienda y comparten habitación con tres; 61,01% presentaban peso por encima del normal y 23,16% tenían al menos una comorbilidad de riesgo para complicaciones por COVID-19 (Tabla 1). Durante la captación los participantes se encontraban afebriles (mediana 36,5C^o^, RIQ:36,3-36,6), con pulso y oximetría normales (mediana 80/minuto, RIQ:74-87; mediana 98%, RIQ:97-99).

**Tabla 1:** Características demográficas y antecedentes de los participantes del estudio (n=462)

Característica	n	Porcentaje
Edad^a^	460	30,81 (25,35 - 39,30)
Sexo Femenino	434	93,98
País de nacimiento		
Venezuela	459	99,35
Colombia	3	0,65
Seguridad Social		
Sin seguro/no regularizado	329	71,21
Subsidiado	54	11,69
Vinculado (SISBEN)	36	7,79
Regularizado/sin seguro	28	6,06
Contributivo	14	3,03
Prefiero no responder	1	0,22
Años de estudios^a^	461	11 (9-11)
Nivel educativo		
Analfabeta	6	1,30
Primaria incompleta	11	2,38
Primaria completa	24	5,19
Secundaria incompleta	121	26,19
Secundaria completa	182	39,39
Tecnología incompleta	3	0,65
Tecnología completa	33	7,14
Universitario incompleto	33	7,14
Universitario completo	47	10,17
Posgrado	2	0,43
Ocupación		
Ama de casa	208	45,02
Vendedor ambulante	95	20,56
Empleado	66	14,29
Reciclador	8	1,73
Otros trabajadores	56	12,12
Desempleado	24	5,19
Estudiante	3	0,65
No trabaja por condición de salud	1	0,22
Prefiero no responder	1	0,22
Años de estancia en Colombia^a^	462	2,05 (1,26 - 2,86)
Horas en casa^a^	462	23 (12 - 24)
Total de convivientes en casa^a^	462	5 (4 - 7)
Adultos que viven en la casa^a^	462	3 (2 - 5)
Menores de edad que viven en la casa^a^	462	2 (1 - 3)
Personas con quien comparte habitación^a^	462	3 (2 - 4)
Característica	n	Porcentaje
Índice de Masa Corporal (IMC)^a^	336	26,23 (23,44 - 29,90)
Clasificación de IMC	336	
Bajo peso <18,5	15	4,46
Normal 18,8 - <25	116	34,52
Sobrepeso 25 - <30	122	36,31
Obesidad >30	83	24,70
Tabaquismo		
Nunca ha fumado	309	66,88
Actualmente fuma	78	16,88
Dejó de fumar	75	16,23
Comorbilidad		
Sí	153	33,12
No	302	65,37
No sabe	7	1,52
Enfermedad de la tiroides	8	1,73
Alergias	7	1,52
Enfermedades ácido-pépticas	6	1,30
Comorbilidad de riesgo para COVID-19^b^		
Ninguna	355	76,84
Una	98	21,21
Dos	7	1,52
Tres	2	0,43

*a Mediana (RIQ).b Asma (12,99%), hipertensión (8,23%), diabetes (2,16%) y enfermedad renal (1,08%). Cáncer, hepatopatía crónica, EPOC y accidentes cerebrovasculares no fueron reportados.*

*Otro síntoma: se encuentra la congestión nasal (5), el malestar general (2), y dolor de oído, vómito, carraspeo, conjuntivitis, dolor articular, vómito y mareo que fueron referidos cada uno por un participante.*

**Figura 1:**
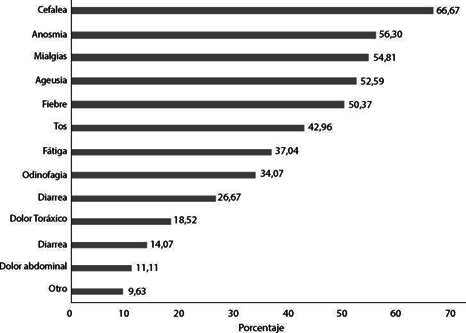
Frecuencia de los síntomas sugestivos de COVID-19 en los migrantes que refirieron al menos un síntoma desde el inicio de la pandemia (n=135).

De los participantes, el 29,22% (135) tuvo al menos un síntoma de COVID-19. La mediana de meses desde el inicio de síntomas hasta la encuesta fue de 4,14 (n=128, RIQ:1,51-7,00). La cefalea fue el síntoma más frecuente, seguida de anosmia, mialgias, ageusia y fiebre (Figura 1). Solo 2,96% (4/135) tuvieron acceso a pruebas diagnósticas (3 serológica y 1 RT-PCR) y 4,44% (6/135) consultaron al médico, ninguno estuvo hospitalizado, aunque uno requirió oxígeno durante la atención por urgencias.

En cuanto al contacto con alguna persona con sospecha/diagnóstico de COVID-19, 19,70% lo reportaron; 47,25% contestó que ni el caso ni el participante estaban utilizando tapabocas durante el contacto, y la mayoría habían estado a menos de dos metros de distancia, en un lugar cerrado y por más de 15 minutos (Tabla 2). Respecto a las prácticas relacionadas con transmisión de SARS-CoV-2, más del 90% contestaron que se lavaban las manos cada vez que podían y que usaban tapabocas; 17,10% utilizaban otras medidas como guantes (9,52%), caretas (3,90%), alcohol/gel (3,03%) y gorro/ bata (1,30%). Además, 60% asistió durante la pandemia a algún evento de riesgo; ninguno viajó en avión, pero 18,40% tuvo movilidad nacional o internacional. Adicionalmente, 53,90% utilizaba un medio de transporte seguro (pie/bicicleta/transporte propio) (Tabla 3).

**Tabla 2:** Caracterización del contacto con casos sospechosos o confirmados de COVID-19

Característica	n	%
Contacto con alguien sospechoso/confirmado	462	
No	364	78,79
Sí, con alguien sospechoso	56	12,12
Sí, con alguien confirmado	35	7,58
No sabe	7	1,52
Relación con el caso sospechoso/confirmado de COVID-19	91	
Conviviente	50	54,95
Compañero de trabajo	16	17,58
Otra persona	14	15,38
Vecino	8	8,79
No sabe/No responde	3	3,30
Tiempo desde el contacto hasta la encuesta	91	
Todos los días	22	24,18
Una semana	15	16,48
8 días y un mes	10	10,99
Más de un mes	42	46,15
No sabe/No responde	2	2,20
Uso del tapabocas durante el contacto	91	
Ninguno con tapabocas	43	47,25
Ambos con tapabocas	35	38,46
Participante con tapabocas	9	9,89
No recuerda/sin dato	3	3,30
Enfermo con tapabocas	1	1,10
Contacto a menos de dos metros	91	
Sí	73	80,22
No	15	16,48
No recuerda/sin dato	3	3,30
Característica	n	%
Contacto en lugar cerrado	91	
Sí	75	82,42
No	13	14,29
No recuerda/sin dato	3	3,30
Tiempo del contacto	91	
Menos de 5 minutos	4	4,40
5 - 15 minutos	10	10,99
30 - 60 minutos	11	12,09
Más de una hora	61	67,03
No recuerda/sin dato	5	5,49

**Tabla 3:** Prácticas de los migrantes incluidos en el estudio relacionadas con la transmisión de SARS-CoV-2

Característica	n	%
Lavado de manos		
Cada vez que puedo	423	91,56
Siento las manos sucias	22	4,76
Pocas veces en el día	12	2,60
Rara vez lo hago	5	1,08
Uso del tapabocas		
Siempre que salgo	442	95,67
La mayoría de las veces al salir	12	2,60
Algunas veces al salir	5	1,08
Pocas veces al salir	1	0,22
Nunca	2	0,43
Uso de otra medida de protección	79	17,10
Asistencia a evento de riesgo^a^		
Sí	277	59,96
No	13	2,81
Prefiere no responder	172	37,23
Visita a hospital o centro de salud	210	45,45
Asistencia a alguna iglesia	77	16,67
Viaje bus intermunicipal	45	9,74
Actividades culturales de >50 personas	6	1,30
Manifestaciones/eventos políticos	3	0,65
Viaje		
No	377	81,60
Internacional	46	9,96
Nacional	39	8,44
Medio de transporte utilizado		
A pie/bicicleta	217	46,97
Característica	n	%
Público - bus	114	24,68
Mototaxi	64	13,85
Público - taxi/plataformas tecnológicas	35	7,58
Moto/Carro propio	32	6,93

*a Asistencia, desde que inició la pandemia, a hospital o centro de salud, iglesias, actos políticos o manifestaciones, actividades culturales de más de 50 personas, viajes en avión o viajes intermunicipales en bus.*

**Figura 2:**
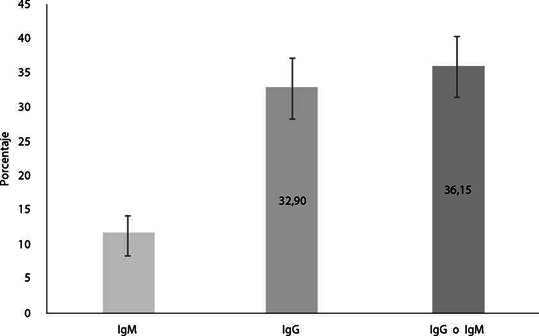
Seroprevalencia de anticuerpos contra SARS-CoV-2 en los migrantes con vocación de permanencia en Bucaramanga, Santander (n=462).

De los participantes, 24,46% (113) fueron positivos solo para IgG considerándose que estuvieron expuestos a SARS-CoV-2 en algún momento desde que inició la pandemia, 8,44% (39) fueron positivos para las dos inmunoglobulinas, interpretándose como infecciones recientes por SARS-CoV-2; y 3,25% (15) fueron solamente IgM positivos, esto podría interpretarse como infecciones muy recientes o como falsos positivos^17^. La seroprevalencia de IgM contra SARS-CoV-2 fue de 11,69% (IC95% 9,07 14,94), la de IgG fue de 32,90% (IC95% 28,77-37,31) y considerando IgM o IgG fue de 36,15% (IC95% 31,89-40,63) (Figura 2). La frecuencia de infecciones asintomáticas fue de al menos 59,88% (IgG o IgM positiva; 100/167), considerando que los síntomas referidos por los sintomáticos positivos fueron causados por infección con SARS-CoV-2.

*La seroprevalencia de IgM ajustada por la sensibilidad (77,5%) y la especificidad (97,5%) de la técnica es de 12,27%. La seroprevalencia de IgG ajustada por la sensibilidad (87,5%) y la especificidad (97,3%) de la técnica es de 35,61%. Se utilizó la fórmula de Sempos y Tian*^*15*^*.*

Se encontró que tener una estancia en Colombia de dos o más años, así como convivir en una casa con seis o más personas, haber tenido contacto con sospechosos/confirmados de COVID-19, haber presentado síntomas de COVID-19 hacía seis meses o más, y la presencia de cada síntoma se asociaron con la seroprevalencia de IgG contra SARS-CoV-2. La anosmia y ageusia fueron los síntomas con mayor magnitud de asociación (RP:>2) (Tabla 4).

**Tabla 4:** Características asociadas a la seroprevalencia de IgG contra SARS-CoV-2 en migrantes con vocación de permanencia en Bucaramanga.

Característica	Seronegativo	Seropositivo		RP^a^	IC95%^b^
	310 (67,10%)	152 (32,90%)	^*p*^		
Estancia en Colombia (años)^c^	1,96 (1,21 - 2,81)	2,23 (1,39 - 2,99)	0,0133	1,04	0,97 - 1,12
Estancia en Colombia n (%)			0,006		
Menor de 2 años	159 (51,29)	57 (37,50)		Ref.	
Mayor o igual a 2 años	151 (48,71)	95 (62,50)		1,43	1,11 - 1,92
Convivientes en casa			0,05		
1-3	77 (24,84)	26 (17,11)		Ref.	
4-5	101 (32,58)	44 (28,95)		1,20	0,79 - 1,82
6 o más convivientes	132 (42,58)	82 (53,95)		1,52	1,05 - 2,20
Contacto con caso COVID-19			0,027		
sospechoso/confirmado					
No	256 (82,58)	108 (71,05)		Ref.	
Si, con alguien sospechoso	30 (9,68)	26 (17,11)		1,56	1,13 - 2,16
Si, con alguien confirmado	19 (6,13)	16 (10,53)		1,54	1,04 - 2,29
No sabe	5 (1,61)	2 (1,32)		0,96	0,30 - 3,14
Síntomas de COVID-19	74 (23,87)	61 (40,13)	<0,001	1,62	1,26 - 2,10
Anosmia	28 (9,03)	48 (31,58)	<0,001	2,34	1,85 - 2,97
Ageusia	27 (8,71)	44 (28,95)	<0,001	2,24	1,76 - 2,86
Cefalea	49 (15,81)	41 (26,97)	0,006	1,53	1,16 - 2,01
Mialgias	35 (11,29)	39 (25,66)	<0,001	1,81	1,39 - 2,36
Fiebre	33 (10,65)	35 (23,03)	0,001	1,73	1,31 - 2,29
Tos	30 (9,68)	28 (18,42)	0,011	1,57	1,16 - 2,13
Fatiga	23 (7,42)	27 (17,76)	0,001	1,78	1,33 - 2,39
Disnea	18 (5,81)	18 (11,84)	0,027	1,59	1,11 - 2,27
Diarrea	8 (2,58)	11 (7,24)	0,024	1,82	1,21 - 2,73
Meses desde que presentó síntomas*	n=71	n=57	0,05	1,05	0,99 - 1,11
	3,25 (1,25 -5,88)	4,90 (1,87 - 7,40)			
Meses desde inicio síntomas			0,001		
Ningún síntoma	237 (77,20)	91 (61,49)		Ref.	
< 2 meses	24 (7,82)	15 (10,14)		1,39	0,90 - 2,14
2 meses - < 6 meses	30 (9,77)	20 (13,51)		1,44	0,98 - 2,11
6 meses o más	16 (5,21)	22 (14,86)		2,09	1,51 - 2,88

*aRP: Razón de Prevalencia. bIC95%: Intervalo de Confianza de 95%. cMediana (RIQ).*

## Discusión

Los migrantes con vocación de permanencia participantes en el estudio en su mayoría son mujeres residentes en Colombia desde hace menos de tres años. Cerca de un cuarto tienen comorbilidades y más de la mitad tienen sobrepeso u obesidad, todos estos son factores relacionados con la gravedad de COVID-19. Aunque la mayoría realizaban higiene de manos frecuente y usaban tapabocas al salir de sus viviendas, acciones que se recomiendan para disminuir el riesgo de infección, la quinta parte tuvieron contacto con un caso sospechoso/confirmado de COVID-19, siendo la mayoría de alto riesgo de transmisión de SARS-CoV-2 puesto que estaban sin tapabocas, sin distanciamiento físico, en un lugar cerrado y fue por más de 30 minutos.

La seroprevalencia para IgG/IgM encontrada en el presente estudio fue similar a la reportada en trabajadores migrantes, hombres, con al menos seis meses de residencia en Kuwait, entre mayo y junio de 2020 (IgG/IgM: 38,1%, n=525^8)^. También, fue similar a la seroprevalencia reportada en la línea base (anticuerpos neutralizantes: 30,4%, n=478) de un estudio en Singapur realizado entre mayo y julio 2020, que incluyó hombres adultos, inmigrantes y trabajadores residentes en dormitorios. Sin embargo, la seroprevalencia en esta población aumentó hasta 63,8% en el seguimiento a seis semanas^18^. En contra posición, la seroprevalencia encontrada en el presente estudio fue menor a la reportada en París en hombres migrantes trabajadores, residentes en dos refugios de Médicos sin Fronteras entre junio y julio de 2020 (IgG: 88,7%, n=124^9)^, y en Singapur (60,1%, n=135.760), en hombres trabajadores residentes en edificios-dormitorios entre marzo y julio de 2020^10^.

Por el contrario, la seroprevalencia en los migrantes con vocación de permanencia estudiados fue mayor a la reportada en tres estudios^19^^,^^20^^,^^21^. El primero realizado en la provincia de Hai Duong, Vietnam, entre enero y febrero de 2021, que incluyó un subgrupo de trabajadores migrantes, sintomáticos y asintomáticos, que se encontraban en dos instalaciones de cuarentena por tener contacto con convivientes con COVID-19, y que reportó una seroprevalencia de 0,47% (1/21219). El segundo estudio realizado en Francia, que incluyó población migrante de primera generación nacidos en Europa y fuera de Europa, encontró una seroprevalencia en mayo de 2020 de 3,8% y 9,2%, respectivamente en estas subpoblaciones. Además, para noviembre de 2020, la seroprevalencia aumentó a 5,2% en los migrantes nacidos en Europa y a 13,3% en los nacidos fuera de Europa^20^. Y el tercer estudio, realizado en Omán entre julio y noviembre de 2020 en población asintomática mayor de 4 años, que reportó una seroprevalencia en migrantes de 9,1% en la evaluación basal, la cual aumentó a 16,8% entre 8 y 16 semanas más tarde^21^.

Por otra parte, la seroprevalencia encontrada fue similar a la reportada para Bucaramanga en el Estudio País realizado por el Instituto Nacional de Salud (32% IC95% 29%-36%^22)^, pero mayor a la reportada en trabajadores del Área Metropolitana de Bucaramanga (AMB) (19,5%) donde 59,9% eran mujeres^23^. Esta diferencia podría explicarse parcialmente porque el muestreo de este estudio fue anterior (septiembre-diciembre/2020).

En resumen, estas diferencias en la seroprevalencia reportada en los estudios podrían explicarse por las condiciones disímiles de sexo, residencia concentrada en lugares específicos y del comportamiento epidemiológico de la Pandemia de COVID-19 en las ciudades estudiadas. Adicionalmente, aunque en menor medida que por las características de las poblaciones, las diferencias pueden ser parcialmente explicadas por el rendimiento de la prueba diagnóstica utilizada, porque se reconoce que, especialmente la sensibilidad, varía dependiendo del método de la prueba^24^.

En cuanto a los factores asociados a la seropositividad, el contacto con un caso sospechoso/confirmado de COVID-19 se asoció a este desenlace similar a lo reportado en trabajadores del AMB^23^; sin embargo, en migrantes en Paris no se encontró esta asociación^9^, aunque si evidenciaron asociación positiva entre la seroprevalencia y la cantidad de personas que compartían habitación, baño o cocina^9^, similar a lo observado en el presente estudio para la cantidad de convivientes. Adicionalmente, en migrantes se ha reportado que el tabaquismo estuvo asociado a menor seroprevalencia^8^^,^^9^, sin embargo, no evidenciamos esta asociación, como tampoco fue reportada en Singapur^18^.

## Conclusiones

En migrantes con vocación de permanencia la seroprevalencia contra SARS-CoV-2 fue similar a la reportada en residentes de Bucaramanga, y al menos 60% de las infecciones fueron asintomáticas. El contacto con casos sospechosos/confirmados de COVID-19, así como las condiciones de hacinamiento, y el tiempo de residencia fueron algunos de los factores asociados a la seroprevalencia.
